# Personal data store ecosystems in health and social care

**DOI:** 10.3389/fpubh.2024.1348044

**Published:** 2024-02-07

**Authors:** Laura Carmichael, Wendy Hall, Michael Boniface

**Affiliations:** ^1^IT Innovation Centre Part of the Digital Health and Biomedical Engineering Research Group, School of Electronics and Computer Science, University of Southampton, Southampton, United Kingdom; ^2^School of Electronics and Computer Science, Web Science Institute, University of Southampton, Southampton, United Kingdom

**Keywords:** data donation, data governance, data portability, personal data store ecosystems, personal data sovereignty, privacy-by-design, self-managing data

## Abstract

This paper considers how the development of personal data store ecosystems in health and social care may offer one person-centered approach to improving the ways in which individual generated and gathered data—e.g., from wearables and other personal monitoring and tracking devices—can be used for wellbeing, individual care, and research. Personal data stores aim to provide safe and secure digital spaces that enable people to self-manage, use, and share personal data with others in a way that aligns with their individual needs and preferences. A key motivation for personal data stores is to give an individual more access and meaningful control over their personal data, and greater visibility over how it is used by others. This commentary discusses meanings and motivations behind the personal data store concept—examples are provided to illustrate the opportunities such ecosystems can offer in health and social care, and associated research and implementation challenges are also examined.

## 1 Introduction

The wide-spread availability and use of wearables and other personal monitoring and tracking devices for health and wellness has increased opportunities for people to gather, collect, generate, and analyze data about themselves. Some examples being “personal genomics testing; diagnostic apps; and fitness, diet, and menstrual trackers” (1), which can be viewed as generating types of “participatory personal data” (2). There are various reasons why an individual may decide or may be compelled, to gather and generate data. For example, in some cases, a person may choose to do this for their own purposes (e.g., to work toward certain fitness goals), and then elect to share their data with peers for support (3). In other cases, a person may be expected or incentivized by others (e.g., insurers, clinicians) to engage in such activities—for instance, as part of a medical rehabilitation program (3). Of course, it must be emphasized that not everyone makes the choice to actively collect, generate, or gather such data about themselves—or even has access to, or the necessary support to effectively use such technologies. However, the amount and variety of individual-generated data is only expected to grow, especially with increasing cyber-physical integration across all sectors of society [e.g., (4, 5)]—e.g., consider the potential for an increasing number of “smart monitoring devices […] built into everyday appliances” (6), or for a majority of smartphones to feature inbuilt sensors and automatically installed health applications (7).

Yet, for many “individuals,” “health systems” and “researchers,” these person-generated data are often “not readily accessible” (8)—despite the right to data portability, which has been under-utilized in practice [see (9, 10)]. Attention is on how data practices across health and social care can be advanced to make better usage not only of traditional data flows, but also those emerging from this progressive digitalization and datafication of health (11, 12). Enabling individuals “to contribute personal data” safely and securely, where they should want to do so, is recognized as an important aspect of “secondary use of health data” that encourages “patient and public participation” (13). For example, approaches are needed that can better facilitate the sharing of “individual-generated data from monitors, wearables and trackers” with clinicians for individual care and with researchers (14).

In this paper, we specifically consider one type of socio-technical innovation: the development of trustworthy personal data store ecosystems, as a person-centered approach for helping to improve the use of individual generated and gathered data for wellbeing, individual care, and research. First, we discuss meanings and motivations for using personal data stores. Second, we outline examples to illustrate some of the opportunities personal data store ecosystems can offer in health and social care. Third, we explore research and implementation challenges associated with the development of such ecosystems. This commentary aims to prompt further conversation around the implications of developing trustworthy personal data ecosystems in health and social care—specifically the extent to which such an approach may contribute to more participatory data practices [e.g., see (12, 15)]—and to encourage further research and development in this area.

## 2 What are personal data stores?

Personal data stores can be viewed as a type of privacy enhancing technology (16, 17) that aim to provide safe and secure digital spaces, which enable people to self-manage, use, and share their personal data with others in a way that aligns with their individual needs and preferences (18). Effective and appropriate data controls and services are required [e.g., see (19)] so that a personal data store can be used by an individual for self-managing their personal data for their own personal use (e.g., for personal data generation, data portability, personal analytics, data retention, data deletion), and for consenting to secondary use by others (e.g., for permissioning, data access, monitoring re-usage, de-identification).

### 2.1 Motivations

In the “current attention economy” (20), conventional data models and practices are typically “organization-centric” (21)—in that, much of the personal data (e.g., behavioral data) that people generate when online is collected, held and exploited by big data platforms in ways often invisible to them (20). Whereas personal data store ecosystems assume the form of more person-centered data models and practices [e.g., (21)], aiming to foster greater “personal data sovereignty” (22–24)—that is, giving individuals more access and control over their personal data, and greater visibility over how it is used by others. Such individual control should be “meaningful” (19) in the sense that people are able to determine how their personal data (as part of their personal data stores) can be accessed and used by others within safe and secure data ecosystems, and how such secondary uses may be of benefit to themselves and others. Seeing that individuals are increasingly becoming both active consumers and producers of personal data (25), the personal data store approach enables people to be more than passive data subjects (5). The expectation being that greater use of personal data stores will incentivize the development of new data-related services for users and encourage people to share more data for individual and societal benefit (22, 23).

Despite a diverse range of personal data store initiatives—e.g., BBC Box,^1^ Hub of All Things,^2^ Mydex,^3^ Solid^4^—personal data stores are yet to be widely adopted and used. However, the development of data spaces, marketplaces and other innovative approaches to personal data sovereignty remains a key area of interest for governments, industry and the public sector. For instance, giving individuals greater control over their personal data is one of the ambitions for the proposed EU European Health Data Space Regulation (26, 27); and for smart data schemes development in terms of consumer data [see, e.g., (28)].

## 3 Using personal data stores in health and social care

Four illustrative examples of how personal data stores might be utilized to help improve the ways in which individual-generated data can be used for wellbeing, individual care and research are now presented:

- Using data within **health and wellbeing apps**.
- Using data for **individual care**.
- **Donating data** for health and social care research.
- **Participating** in health and social care research.

These examples are by no means exhaustive but serve to highlight some of the opportunities that personal data stores can offer in the context of health and social care.

### 3.1 Using data within health and wellbeing apps

Personal health data are already being accessed, collected and generated by many individuals through different types of task specific apps for health and wellness. This includes those offered by healthcare providers (e.g., see NHS App,^5^ My Medical Record^6^) and others provided by technology companies (e.g., fitness trackers). People may want to use their personal data stores to bring data from different health networks, apps and devices together in one place and to use their data in accordance with their individual needs and preferences. Given that people often switch between apps, devices and technology providers, the use of personal data stores for data archival can also help to ensure that people do not lose access to data as technologies are replaced or become non-operational or obsolete [e.g., (29)]. Such a user-centric approach (22, 23) to data access, management and reuse may also help to drive the creation of and connection to other health and wellbeing services (e.g., for individual care, data donation) and increase the usability of existing apps (e.g., through enhanced mechanisms for data portability).

### 3.2 Using data for individual care

Personal data stores can provide one way in which people can share a wide-range of individual-generated data with health and social care professionals responsible for their care (30). By way of illustration, such services may provide patients with digital tools for “automatic form filling” and for communication of their “health story” with those responsible for their care to avoid unnecessary repetition (30).

### 3.3 Donating data for health and social care research

Many people are willing to contribute to health-related research by actively volunteering to make their personal data available [e.g., from wearable devices, smartphone apps (31)] for specified research purposes where there is expected public benefit [e.g., see (32, 33)]. Examples being the UK Biobank^7^ and Open Humans^8^ [see (8, 15)]. Such data donation activities have also been vital in the response to the COVID-19 pandemic—as illustrated by the many people who voluntarily contributed their data to COVID-19 surveillance research studies (e.g., via the Corona Datenspende App,^9^ DETECT study,^10^ Zoe Health Study App^11^). People may want to use their personal data stores as a means to donate their personal data from different health networks, apps and devices for use as part of health and social care research.

### 3.4 Participating in health and social care research

In many cases, there can be limited direct interaction, or absence thereof, between those donating data and researchers in the data analysis process following an act of data donation (34). However, people can participate in research in ways beyond their passive involvement as a data subject (35)—for example, as co-designers or as active non-professional researchers as part of citizen science initiatives [e.g., (36)]. Personal data stores may also offer people the opportunity to “bring your own data” [e.g., (37)], as part of their active participation in health and social care research studies [see (34)]—where it would be considered safe, secure and appropriate (e.g., lawful, ethical) to do so. For instance, seeing that personal data represent a fundamental aspect of personal health technologies (e.g., wearable devices, health apps), such participatory data analysis may help to enrich research studies on usability testing of such technologies [e.g., (38)]. It may also help with patient and public involvement [e.g., see (39)] related to the use of advanced analytics in health—e.g., machine learning models for glucose prediction in real-time could be explained to people with type 1 diabetes through an interactive computational notebook, enabling participants and researchers to explore these models together using real-world data as part of co-design sessions [example based on: (40)].

## 4 Challenges of developing personal data store ecosystems

Significant care is required to develop trustworthy personal data store ecosystems that are secure, rights-respecting and supported by a wide range of stakeholders—e.g., patients, clinicians, researchers. To improve the use of data for wellbeing, individual care and research, it is crucial that such trustworthy ecosystems can handle different types of stakeholder interactions and provide services that “pass the test of convenience” (41) for individuals and other users. Enabling more person-centered approaches to data sharing in health and social care in practice requires consideration of a wide range of factors—such as, issues related to ethical and legal compliance, standardization and determination of appropriate business models [e.g., see (18, 42–44)]. By way of illustration, in this paper, we specifically focus attention on three areas of socio-technical research and implementation challenges:

- People being able to **self-manage** their personal data safely, securely, and appropriately.
- All stakeholders committing to the **data work** needed to extract value from data.
- Stakeholders being able to work together to establish the **sustainable infrastructure** necessary for supporting and governing such ecosystems.

### 4.1 Challenge of self-managing data

Much has been written about the various issues surrounding the notion of self-managing personal data where individuals consent to secondary uses of their data [e.g., see (45–49)]. For instance, one issue is that while many people often want more control over their personal data, in practice the majority of people do not read privacy notices—or when they do, may not have enough further information and knowledge to make informed decisions over personal data (48). It should also be highlighted that people may not always follow best practices for cybersecurity [e.g., see (50)]. Another issue is that personal data often conveys information about our interactions or relationships with one or more other people (46, 51, 52), decisions to self-manage and share personal data therefore often pose privacy and security risks not only to the personal data store user, but also to those people that they are associated with.

Usage of personal data should also be considered in the context of the existing health app-centric ecosystem—where individuals are often not only motivated to generate and collect progressively more and diverse types of (sensitive) personal data but are also encouraged to share their data with peers and other third parties [e.g., (1, 53)]. In some cases, this may be problematic as individuals may be making available excessive amounts of data about themselves (and others) either intentionally or inadvertently, which may give rise to increased privacy and security risks. As data-rich environments offering people access to extensive archives of personal data (e.g., from multiple health networks, apps, and devices), thought needs to be given to the impact personal data store usage could have on such possible excessive data sharing.

The ability of people to self-manage their personal data safely, securely, and appropriately, and their willingness to self-manage data, together pose a significant challenge to the development of personal data store ecosystems (18). While there is no simple solution to this problem, service providers need to develop solutions in accordance with privacy-by-design [see (44, 54)], ensuring that effective mechanisms are in place that can keep data safe and secure by default in multi-stakeholder personal data store ecosystems—and individuals and other stakeholders are supported and encouraged to comply with best practices for privacy, data protection and security across the data lifecycle [e.g., see (50, 55)]. Examples of how personal information management systems “can support data protection principles” in practice are given in Opinion 9/2016 of the European Data Protection Supervisor (44)—such as, mechanisms for partially automated “consent management” [also see (56) for discussion about privacy preference recommendation systems]; “data security” including, “encryption” and “identification” and “authorization” measures; “traceability” [also see (57, 58) for related discussion on assurance], and “transparency” [also see (24) for discussion of “machine-readable policies”].

### 4.2 Challenge of work needed to extract value from data

A considerable amount of time and effort is likely to be required to carry out the various forms of “data work” [see (59, 60)] necessary to make data accumulated in personal data stores meaningful and useful for individuals and other users (e.g., in terms of wellbeing, individual care, research). Self-management of personal data is one essential component of overall data work required—however, other important functions carried out by service providers adding value to data must also be recognized (22). It should further be noted that by identifying the various forms of data work involved, a better understanding of where such activities may have adverse implications for stakeholders and what measures can be taken to improve the given situation can be achieved.

Of course, in some cases, people will not be in the position to self-manage their personal data, or do not want the responsibility of doing so (61). Consideration also must be given to those situations where personal data store ecosystems may have adverse implications for stakeholders, such as where their application in specific contexts would be regarded as: unduly burdensome, e.g., self-managing data seen as contributing to “illness work” by patients [e.g., (62)]; inappropriate, e.g., as part of participatory research analysis due to ethical sensitivities; or would contribute to, or otherwise expand, existing health inequalities and digital exclusion (61).

Ensuring that services for data sharing in personal data store ecosystems are perceived as trustworthy, convenient and valuable by stakeholders [e.g., (41, 63)]—e.g., in helping them to achieve their aims in ways which align with their needs and preferences—is essential for encouraging greater use of personal data stores. Engaging stakeholders in the co-design of such services is therefore crucial for their development and validation [e.g., (30, 63)] as well as for understanding stakeholder incentives, expectations and concerns associated with person-centered data sharing approaches.

### 4.3 Challenge of establishing sustainable infrastructure

Establishing the socio-technical infrastructure necessary to effectively govern, operate, and support the use of multi-stakeholder personal data ecosystems in health and social care will be challenging [e.g., (17, 22, 41, 42)]. Such infrastructure needs to enable data portability between multiple personal data store providers as well as other service providers (e.g., as individuals may decide to switch provider), and interoperability to deliver the services required to support the different uses of personal data stores in health and social care [e.g., (44)]. Further, such infrastructure should facilitate “participatory data stewardship” (15) not only on the account of individuals self-managing their data as part of personal data stores, but also by mechanisms that allow individuals to influence and contribute to how data are governed once made available to others [e.g., for individual care, research] in different “data governance spaces” (64). As personal data stores ecosystems need to appropriately balance risks and benefits at both “individual” and “population” levels (65), attention needs to be given on the types of trusted third-party intermediaries (65) that are required to give rise to robust data stewardship practices across multi-stakeholder ecosystems—e.g., “consent intermediaries” (46) [for further examples also see (57, 66, 67)].

To establish the necessary infrastructure, there needs to be focus on “whole systems” (30) as well as how to incentivize [e.g., (44)] support and participation from a wide range of stakeholders. However, efforts are being made to move toward such infrastructure, e.g., with the proposed development of the European Health Data Space as well as the focus on smart data initiatives (28) and deployment of personal data stores in other domains [e.g., (43)].

## 5 Conclusion

Against the backdrop of progressive digitalization and datafication of health, we have specifically focused on the development of trustworthy personal data store ecosystems—as one type of socio-technical innovation—providing a person-centered approach that could help to improve the use of data for wellbeing, individual care and research. As we have highlighted, various opportunities for using personal data stores exist in health and social care, such as for enabling individuals to make better use of their data within health and wellbeing apps, use data for individual care, donate data, and participate in health and social care research. Yet, developing the trustworthy personal data ecosystems needed to support such beneficial uses arising through these different types of stakeholder interactions will be challenging, requiring consideration of multiple factors (e.g., ethical, legal, social, technical, economic) beyond those discussed in this paper. In this commentary, we have focused on three such areas of research and implementation challenges related to the ability and willingness of individuals to self-manage their data; committing to required data work; and establishing sustainable infrastructure. How to ensure that personal data stores can give rise to improved personal data sovereignty through convenient access to data and services in practice, and lead to greater trustworthy data sharing as part of sustainable, secure and rights-respecting ecosystems supported by stakeholders, remains a crucial issue. What is clear from this discussion on motivations, opportunities and challenges is that encouraging wider adoption and use of personal data stores in health and social care calls for a deep understanding of the human factors involved.

## Data availability statement

The original contributions presented in the study are included in the article/supplementary material, further inquiries can be directed to the corresponding author.

## Author contributions

LC: Conceptualization, Writing – original draft, Writing – review & editing. WH: Conceptualization, Funding acquisition, Writing – review & editing. MB: Conceptualization, Funding acquisition, Supervision, Writing – review & editing.

## Footnotes

^1^Available at: https://www.bbc.co.uk/rd/projects/databox (Accessed October 3, 2023).

^2^Available at: https://www.hubofallthings.com/ (Accessed October 3, 2023).

^3^Available at: https://mydex.org/ (Accessed October 3, 2023).

^4^Available at: https://www.inrupt.com/solid (Accessed October 3, 2023).

^5^Available at: https://www.nhs.uk/nhs-app/ (Accessed October 6, 2023).

^6^Available at: https://www.uhs.nhs.uk/for-patients/my-medical-record (Accessed October 6, 2023).

^7^Available at: https://www.ukbiobank.ac.uk/ (Accessed October 6, 2023).

^8^Available at: https://www.openhumans.org/ (Accessed October 6, 2023).

^9^Available at: https://corona-datenspende.github.io/en/ (Accessed February 5, 2024).

^10^Available at: https://detectstudy.org/ (Accessed October 6, 2023).

^11^Available at: https://health-study.zoe.com/data (Accessed October 6, 2023).
